# Species composition in the *Balanophora laxiflora* species complex (Balanophoraceae): molecular and morphological evidence

**DOI:** 10.7717/peerj.21913

**Published:** 2026-11-02

**Authors:** Alexander E. Zavialov, Tahir H. Samigullin, Dmitry F. Lyskov, Constantin I. Fomichev, Maria D. Logacheva, Maxim S. Nuraliev

**Affiliations:** 1 Department of Higher Plants, Faculty of Biology, M.V. Lomonosov Moscow State University, Moscow, Russia; 2 Department of Evolutionary Biochemistry, A.N. Belozersky Institute of Physico-Chemical Biology, M.V. Lomonosov Moscow State University, Moscow, Russia; 3 Joint Vietnam-Russia Tropical Science and Technology Research Center, A.N. Severtsov Institute of Ecology and Evolution, Russian Academy of Sciences, Moscow, Russia; 4 Center for Bio- and Medical Technology, Moscow, Russia; 5 Laboratory of Plant Genomics, Vavilov Institute of General Genetics, Russian Academy of Sciences, Moscow, Russia

**Keywords:** Indochinese Peninsula, Parasitic plants, Phylogeny, Santalales, Vietnam

## Abstract

The *Balanophora laxiflora* species complex is one of the most taxonomically difficult lineages within the holoparasitic genus *Balanophora*. Over the last century, a number of contrasting opinions have been expressed regarding the identities and relationships of the species currently considered as members of this complex. Although the complex is widely distributed across tropical and subtropical Asia, previous studies have focused on its Japanese and Chinese representatives, whereas populations from Southeast Asia have received little attention. Here, we fill this gap through an integrative study with employment of a set of original specimens from several provinces of Vietnam. We present the results of a comprehensive morphological assessment that includes examination of living plants *in situ* and analysis of alcohol-fixed specimens using light and electron microscopy. We also provide an expanded molecular phylogenetic reconstruction of the species complex and establish the phylogenetic placement of our Vietnamese specimens. We report extensive contradictions between the characteristics of the studied specimens and the presumed morphological boundaries of the currently recognized species. In particular, the dimensions of the claviform bodies of the female inflorescence and their cuticle morphology frequently showed variants attributed to different species within a single specimen. Even when these features were excluded from consideration, 10 of the 16 studied specimens could not be assigned to any species, because each of these specimens demonstrated a mixture of character states considered diagnostic for different species, most of such plants being morphologically intermediate between *B. laxiflora* and *B. yakushimensis*. The phylogenetic results do not contradict the idea of distinctness of *B. appressa*, *B. japonica* and *B. nipponica*. At the same time, *B. laxiflora*,* B. parajaponica* and *B. yakushimensis* were inferred as non-monophyletic, and their monophyly cannot be achieved even if the morphologically intermediate specimens are conventionally assigned to species according to their phylogenetic placement. The morphological and phylogenetic evidence therefore unequivocally indicates the need for re-shaping the interspecific boundaries, with one of the possible decisions being merging of* B. laxiflora*,* B. parajaponica* and *B. yakushimensis* into a single species. Further analysis has shown, however, that even after such a modification, the species in the *B. laxiflora* complex would only weakly be differentiated from each other morphologically. Thus, recognition of the entire lineage as a single species might appear the most appropriate taxonomic solution. We highlight the importance of comprehensive sampling in studies of intricate lineages similar to the *B. laxiflora* species complex, especially broad geographical coverage and careful assessment of structural diversity within each analyzed population and specimen.

## Introduction

*Balanophora* J.R.Forst. & G.Forst. comprises about 25 species, being the largest genus in the family Balanophoraceae (Kuijt & Hansen, 2015; Svetlikova et al., 2026). *Balanophora* is well-known for its outstanding morphological complexity, coupled with exceptionally difficult and controversial taxonomic delimitation (Harms, 1935; Hansen, 1972b; Hansen, 1999; Eberwein, Nickrent & Weber, 2009). The lineage referred to here as the *B. laxiflora* species complex is among the most problematic groups in the genus. This lineage is currently accepted as comprising six species: *B. appressa* R.X.Yu & S.Y.Zhou, *B. japonica* Makino, *B. nipponica* Makino, *B. parajaponica* R.X.Yu, S.Y.Zhou & Y.Q.Li, *B. yakushimensis* Hatus. & Masam., and *B. laxiflora* Hemsl. itself (Yu et al., 2021; Svetlikova et al., 2026), whereas 16 species have been described within the species complex in total (POWO, 2026). The lineage has a broad distribution in the subtropics of East Asia and also extends to the tropical Indochinese Peninsula and Hainan (Hansen, 1972a; Hansen, 1972b; Hansen, 1973; Murata, 1999; Huang & Murata, 2003; Aung et al., 2025).

The most recent global revision of *Balanophora* was carried out by Hansen (1972b), who subdivided the genus into two subgenera, subgen. *Balanophora* (with four sections) and subgen. *Balania* (Tiegh.) Valeton (with two sections). Since Hansen’s study, *B. laxiflora* has been placed in *Balanophora* subgen. *Balanophora* sect. *Polyplethia* (Griff.) B.Hansen, whereas the placement of some other members of the *B. laxiflora* species complex has changed significantly over time.

Species within the *B. laxiflora* complex are discriminated on the basis of the characters of the leaves and female inflorescences, whereas the male inflorescences (of the dioecious species) are considered to have no taxonomic significance (Su, Murata & Hu, 2012; Yu et al., 2021). At the same time, the assessment of specimens of *Balanophora* that include only female inflorescences (either due to incomplete material or due to agamospermic reproductive system of a species) is particularly complicated. A remarkable example is *B. japonica*, which is currently known to be closely related to *B. laxiflora* but was assigned by Hansen (1972b) to *Balanophora* subgen. *Balania* sect. *Neobalania* Setch. Similarly, Hansen (1999) synonymized *B. yakushimensis*, another member of the *B. laxiflora* complex, with *B. fungosa* ssp. *indica* (Arn.) B.Hansen, thus erroneously placing it in *B.* sect. *Balanophora* (although *B. yakushimensis* had already been stated to be close to *B. japonica* in its protologue: Hatusima, 1971). The boundaries of the *B. laxiflora* complex have therefore been established only during the last few decades.

The subdivision of the complex into species is likewise far from being straightforward. For example, *B. nipponica* was included by some authors in *B. japonica* (Ohwi, 1965; Hansen, 1972b), whereas *B. appressa* and *B. parajaponica* were described as recently as in 2021 (Yu et al., 2021). As highlighted by Yu et al. (2021), further efforts are required to evaluate the species composition of this group, and in particular, the circumscription of *B. laxiflora* remains unresolved.

Previous studies focused on the structural diversity and phylogeny of the *B. laxiflora* species complex (Su, Murata & Hu, 2012; Yu et al., 2021; Svetlikova et al., 2026) demonstrate a significant geographical bias, as they are based predominantly on subtropical material and do not include any populations from mainland Southeast Asia. Here, we fill this gap, and expand the sampling for morphological evaluation by including a comprehensive set of specimens from seven provinces of Vietnam, along with two additional samples from China. We present a detailed morphological assessment of the specimens, which was obtained during extensive *in situ* observations of living plants and subsequent laboratory analyses. This approach allowed us to document traits that cannot be studied in fixed collections, such as ovary color and variation among individuals within the same population. We also obtained sequences of the nuclear 18S rRNA gene from five specimens and performed phylogenetic reconstructions that also include all previously studied accessions of the species complex. By integrating morphological and phylogenetic data, we reconsider species delimitation in this lineage and identify the most reliable morphological characters for routine taxonomic work on these species.

## Materials and Methods

### Morphological investigation

For morphological examination, we used 16 samples, all collected by the authors of this paper during fieldwork in 2009–2025 as part of studies of the Joint Vietnam-Russia Tropical Science and Technology Research Center under permits 1037, 2225, 2750, 3302, 3535, 5110, 5464 and 5861. Collection details are provided below under Taxonomic Treatment. Examinations and measurements were conducted on plants fixed and stored in 70% ethanol, using an Olympus BX53 microscope (Olympus, Tokyo, Japan) equipped with an Olympus SC50 digital camera (Olympus, Tokyo, Japan) for observation of small parts of plants. Measurements of the claviform bodies of the female inflorescence were performed using cellSens Entry v.4.4.1 imaging software (Olympus, Tokyo, Japan). In addition, living plants were examined before preparation of each alcohol-fixed specimen, and taxonomically important features (including those revealed by inflorescence dissection) were documented through a series of macrophotographs. These features included the traits of leaf arrangement, the presence of a thickening at leaf base, the shape of the apices of the claviform bodies, and ovary color.

For the five specimens included in our molecular phylogenetic reconstruction, details of the female inflorescences were also studied by means of scanning electron microscopy (SEM). For SEM, ethanol-preserved material was dissected in 70% ethanol under an Olympus SZX7 stereomicroscope (Olympus, Tokyo, Japan), dehydrated through a graded series of ethanol followed by absolute acetone, critical-point dried from liquid CO_2_ using a Hitachi HCP-2 critical-point dryer (Hitachi, Tokyo, Japan), and finally coated with gold and palladium using an Eiko IB-3 ion coater (Eiko Engineering Co. Ltd, Tokyo, Japan). Observations were carried out using a CamScan S2 SEM (Cambridge Instruments, Cambridge, UK), a Quattro S SEM (Thermo Fisher Scientific, Czech Republic), and a JSM-6380LA SEM (JEOL, Tokyo, Japan) at Lomonosov Moscow State University.

On average, five individuals were studied from each sample, but in some cases only one individual, depending on the amount of available material. The shape and size of the claviform bodies and the arrangement of the female flowers were examined in, on average, three inflorescences per specimen, and approximately 15 claviform bodies per inflorescence were analyzed. Cuticle micromorphology was studied using three to five claviform bodies per specimen.

General plant terminology follows Beentje (2016) and Simpson (2019), whereas morphological traits specific to *Balanophora* are termed in accordance with Eberwein, Nickrent & Weber (2009), Hansen (1972b) and Yu et al. (2021).

### Phylogenetic inference

In our phylogenetic reconstructions, we employed the nuclear 18S rRNA gene, as it has previously been used successfully in phylogenetic analyses of *Balanophora* (Su, Murata & Hu, 2012; Yu et al., 2021; Svetlikova et al., 2026). Although the ITS region has also been used in some studies (Su, Murata & Hu, 2012; Yu et al., 2021; Yu et al., 2022; Fu et al., 2025), our preliminary analysis of the dataset of Fu et al. (2025) showed the presence of substantial substitution saturation (according to Xia & Lemey, 2009), and we therefore did not include the ITS region in the phylogenetic analyses.

Five specimens belonging to the *Balanophora laxiflora* species complex, four from Vietnam and one from China, were sequenced *de novo*. Vietnamese material of the species complex is included here in phylogenetic analyses for the first time. The specimens were selected to provide representative coverage of the distribution range of the species complex in Vietnam; in particular, they originate from four provinces representing different regions of northern Vietnam: Northwest, Northeast and North Central Coast. With the inclusion of the Vietnamese material, most of the distribution area of the species complex appears to be covered. Although records from Laos, Myanmar and Thailand are also known, no material from these countries suitable for phylogenetic investigation is currently available. The newly generated sequences were deposited in GenBank under accession numbers PZ049912 –PZ049916 (Table 1).

**Table 1:** Accessions used in the molecular phylogenetic part of the study. Newly generated sequences are shown in boldface. For the accessions generated by Yu et al. (2021), the collector’s numbers provided in the paper differ from those submitted to GenBank. Below the numbers from their paper are applied.

**Species**	**Geographical origin and voucher**	**Source**	**GenBank number for 18S**
*Balanophora appressa*	China, Guangdong, *Yu 005a* (type, male accession, IBSC)	Yu et al. (2021)	MW414649
*Balanophora appressa*	China, Guangdong, *Yu 005b* (type, female accession, IBSC)	Yu et al. (2021)	MW414657
*Balanophora appressa*	China, Guangdong, *Yu 006* (paratype, SYS)	Yu et al. (2021)	MZ048344
*Balanophora japonica*	China, Chongqing, *Yu 011* (SYS)	Yu et al. (2021)	MW414653
*Balanophora japonica*	China, Guangxi, *Yu 012* (SYS)	Yu et al. (2021)	MW414654
*Balanophora japonica*	Japan, Kyushu, *BJAP436_JP26*	Svetlikova et al. (2026)	PZ346200
*Balanophora japonica*	Japan, Kyushu, *BJAP592_JP26*	Svetlikova et al. (2026)	PZ346199
*Balanophora japonica*	Taiwan, Taipei, Mt. Datong, *Su 031* (TAI)	Su, Murata & Hu (2012)	JN392873
*Balanophora japonica*	Taiwan, Taipei, Mt. Syongkongnan, *Su 032*	Su, Murata & Hu (2012)	JN392874
*Balanophora japonica*	Taiwan, Taipei, *BJAP_TW26*	Svetlikova et al. (2026)	PZ346194
*Balanophora laxiflora*	China, Guangxi, *Nuraliev et al. G20* (MW)	This study	**PZ049914**
*Balanophora laxiflora*	China, Hong Kong, *ZHL 0209* (KFBG)	Yu et al. (2021)	MW414647
*Balanophora laxiflora*	China, Hubei, *Yu 013* (SYS)	Yu et al. (2021)	MW414651
*Balanophoralaxiflora*	Taiwan, Miaoli, Mt. Sihmasian, *Su 041*	Su, Murata & Hu (2012)	JN392869
*Balanophoralaxiflora*	Taiwan, Pingtung, Mt. Dahan, *Su 027* (TAI)	Su, Murata & Hu (2012)	JN392872
*Balanophoralaxiflora*	Taiwan, Taipei, Mt. Syongkongnan, *Su 043* (TAI)	Su, Murata & Hu (2012)	JN392870
*Balanophora laxiflora*	Taiwan, Taipei, Mt. Syongkongnan, *Su 044*	Su, Murata & Hu (2012)	JN392871
*Balanophora laxiflora*	Vietnam, Son La, *Nuraliev et al. 2798* (MW)	This study	**PZ049912**
*Balanophora nipponica*	Japan, Honshu, *BNIP_JP26*	Svetlikova et al. (2026)	PZ346201
*Balanophora parajaponica*	China, Guangdong, *Yu 001a* (type, male accession, IBSC)	Yu et al. (2021)	MT498079
*Balanophora parajaponica*	China, Guangdong, *Yu 001b* (type, female accession, IBSC)	Yu et al. (2021)	MW414655
*Balanophora parajaponica*	China, Guangxi, *Yu 003* (paratype, SYS)	Yu et al. (2021)	MT498080
*Balanophora parajaponica*	China, Hainan, *Yu 004* (paratype, SYS)	Yu et al. (2021)	MW414658
*Balanophora parajaponica*	Vietnam, Cao Bang, *Nuraliev et al. 2282* (MW)	This study	**PZ049915**
*Balanophora yakushimensis*	China, Guangdong, *Yu 007a* (SYS)	Yu et al. (2021)	MW414650
*Balanophora yakushimensis*	China, Guangdong, *Yu 007b* (SYS)	Yu et al. (2021)	MW414652
*Balanophora yakushimensis*	China, Guangxi, *Yu 009a* (SYS)	Yu et al. (2021)	MW414648
*Balanophora yakushimensis*	China, Guangxi, *Yu 009b* (SYS)	Yu et al. (2021)	MW414656
*Balanophora yakushimensis*	Japan, Kyushu, *BYAK_JP26*	Svetlikova et al. (2026)	PZ346203
*Balanophorayakushimensis*	Taiwan, Nantou, Mt. Lucyu, *Su 064*	Su, Murata & Hu (2012)	JN392875
*Balanophorayakushimensis*	Taiwan, Nantou, Mt. Shiaochu, *Su 074*	Su, Murata & Hu (2012)	JN392877
*Balanophorayakushimensis*	Taiwan, Taoyuan, Mt. Lala, *Su 063* (TAI)	Su, Murata & Hu (2012)	JN392876
*Balanophora yakushimensis*	Taiwan, Taoyuan, *BYAK_TW26*	Svetlikova et al. (2026)	PZ346197
*Balanophoralaxiflora/yakushimensis*	Vietnam, Lao Cai, *Nuraliev, Lyskov NUR 3771* (MW)	This study	**PZ049913**
*Balanophora parajaponica/yakushimensis*	Vietnam, Thanh Hoa, *Nuraliev NUR 4376* (MW)	This study	**PZ049916**
**Outgroups**			
*Balanophora fungosa* ssp. *fungosa*	Taiwan, Taitong, Guanshan, *Huang s.n.*	Su, Murata & Hu (2012)	JN392868
*Balanophora fungosa* ssp. *indica*	China, Yunnan, *Peng et al. 18661* (HAST)	Su, Murata & Hu (2012)	JN392867

Total genomic DNA was isolated from silica-gel-dried or ethanol-fixed material using a Diamond DNA Plant kit (ABT, Russia) following the manufacturer’s instructions. All specimens except for *Nuraliev et al. 2282* were studied by means of Sanger cycle sequencing. Three pairs of primers designed by Su, Murata & Hu (2012) for amplification of the 18S rRNA gene were used (see Supplementary Files). Polymerase chain reaction (PCR) amplification was performed on a Biometra T3000 Thermocycler (Biometra, Germany) using an Encyclo PCR kit (Evrogen JSC, Russia), as described by Su, Murata & Hu (2012): 94 °C for 3 min, 35 cycles of 94 °C for 50 s, 55 °C for 50 s, 72 °C for 1 min, and a final extension of 72 °C for 3 min. Automated sequencing was performed using Big Dye Terminator version 3.1 for both forward and reverse strands on an ABI Prism 3100-Avant sequencer (Applied Biosystems), using the same set of primers.

For the specimen *Nuraliev et al. 2282*, the 18S rRNA sequence was obtained using next-generation sequencing (NGS). DNA was fragmented using a Covaris M ultrasonicator (Covaris, USA). End repair, adenylation and adapter ligation followed by PCR were performed using the NEBNext Ultra II Sample Prep Kit for Illumina (New England Biolabs, USA) according to the manufacturer’s instructions. Libraries were sequenced using an Illumina NextSeq platform (Illumina, USA) with a read length of 76 bp from each end of the fragment. Reads were cleaned using Trimmomatic (Bolger, Lohse & Usadel, 2014). *De novo* assembly was carried out using the CLC Genomics Workbench v.5.5 software package (Qiagen, Germany).

In addition to the newly generated sequences, all previously published 18S rRNA accessions of the *Balanophora laxiflora* species complex were included in the dataset. In total, the dataset comprises 35 accessions of the species complex. *Balanophora fungosa* ssp. *fungosa* and *B. fungosa* ssp. *indica* were included as outgroup taxa based on the phylogenetic study of Svetlikova et al. (2026). Information on all accessions used in the present study, including voucher details, is summarized in Table 1. DNA sequences were aligned manually using BioEdit (Hall, 1999); gap-rich positions (*i.e.,* those with gaps in more than 50% of accessions) were excluded from the analyses. The alignment is provided in the Supplementary Files.

Phylogenetic analyses were performed using both the maximum-likelihood (ML) method and the Bayesian approach. The ML phylogenetic reconstruction and the approximately unbiased (AU) test (Shimodaira, 2002) were conducted using IQ-TREE 2 (Minh et al., 2020) under the GTR+I model, which was selected as the most appropriate model of nucleotide substitution in the built-in ModelFinder utility (Kalyaanamoorthy et al., 2017) according to the Bayesian information criterion. Internal branch support was assessed using the ultrafast bootstrap approximation (UFBoot) with 10,000 replicates (Hoang et al., 2018). Bayesian inference was performed using MrBayes v.3.2.6 (Ronquist et al., 2012) under the same model (GTR+I). A total of 16 Markov chains were run (comprising four parallel runs of four chains each) for 10,000,000 steps, with every 500th tree sampled. The first 1,000 trees were discarded as burn-in, and the remaining trees were used to construct a majority-rule consensus tree and estimate posterior probabilities for internal branches.

Trees were visualized using TreeView v.1.6.6 (Page, 1996).

## Results

### Morphological investigation

The results of the morphological analysis are presented in Table 2. The most taxonomically controversial specimens are illustrated in detail in Figs. 1 and 2. Cuticle morphology of the claviform bodies, as evaluated using SEM, is summarized in Table 3 and Figs. 1, 3 and 4.

**Table 2:** Studied specimens, their identifications under the narrow species concept (following Su, Murata & Hu, 2012 and Yu et al., 2021) and morphological characteristics. The specimens included in the molecular phylogenetic reconstructions are shown in boldface. An ampersand (&) indicates presence of different variants in a specimen.

Specimen	Identification	Reproductive system	Leaf phyllotaxis	Leaves distantly arranged or subrosulate	Leaves spreading, erect or appressed to stem	Presence of a thickening at leaf base	Shape of apices of claviform bodies	Length of claviform bodies (mm)	Width of claviform bodies (mm)	Arrangement of female flowers	Color of ovary
*Kopylov-Guskov, Severova CIF177* (China, Guangdong)	*B. laxiflora* (but some claviform bodies too short)	Dioecious	Opposite	Distantly arranged	Spreading	No	Truncate	0.830–1.128	0.426–0.556	Mostly on main axis, also on stipes of claviform bodies	Unknown
***Nuraliev et al. G20*** (China, Guangxi) PZ049914	*B. laxiflora* (but some claviform bodies too short, and some too wide)	Unknown	Opposite	Distantly arranged	Erect	Yes	Truncate	0.900–1.300	0.376–0.757	On main axis and on stipes of claviform bodies	Yellow
***Nuraliev et al. 2282*** (Vietnam, Cao Bang) PZ049915	*B. parajaponica* (but some claviform bodies too short and too narrow)	Dioecious	Opposite	Subrosulate	Spreading & erect	No	Truncate	0.512–0.650	0.210–0.596	Mostly on main axis, also on stipes of claviform bodies	Unknown
*Nuraliev et al. 2925* (Vietnam, Ha Giang)	*B. yakushimensis* (but all claviform bodies too long)	Unknown	Opposite	Subrosulate	Spreading	No	Convex	0.950–1.388	0.512–0.739	On main axis and on stipes of claviform bodies	Unknown
*Nuraliev NUR 4239* (Vietnam, Lang Son)	Intermediate between *B. laxiflora* and *B. yakushimensis*	Dioecious	Opposite	Subrosulate	Spreading	No	Truncate	0.700–0.800	0.233–0.390	On main axis and on stipes of claviform bodies	Yellow
*Nuraliev NUR 4244* (Vietnam, Lang Son)	Intermediate between *B. laxiflora* and *B. yakushimensis*	Dioecious	Opposite	Subrosulate	Spreading	No	Truncate	0.400–0.610	0.201–0.366	Mostly on main axis, also on stipes of claviform bodies	Unknown
*Nuraliev 2334* (Vietnam, Lao Cai)	Intermediate between *B. laxiflora* and *B. yakushimensis*	Dioecious	Opposite	Distantly arranged	Spreading & erect	No	Convex	0.718–0.990	0.289–0.613	Mostly on main axis, also on stipes of claviform bodies	Unknown
*Nuraliev, Lyskov NUR 3733* (Vietnam, Lao Cai)	*B. laxiflora* (but claviform bodies as wide as in *B. japonica* and convex as in *B. yakushimensis*)	Unknown	Alternate	Distantly arranged	Erect	Yes	Convex	1.076–1.500	0.793–1.036	Mostly on main axis, also on stipes of claviform bodies	Unknown
***Nuraliev, Lyskov NUR 3771*** (Vietnam, Lao Cai) PZ049913	Intermediate between *B. laxiflora* and *B. yakushimensis*	Unknown	Opposite	Distantly arranged	Spreading & erect	No	Convex & truncate	1.000–1.200	0.414–0.820	Mostly on main axis, also on stipes of claviform bodies	Unknown
*Nuraliev NUR 179a* (Vietnam, Lao Cai)	Intermediate between *B. japonica* and *B. laxiflora*	Unknown	Opposite	Subrosulate	Erect	No	Truncate	0.853–1.008	0.195–0.473	Mostly on main axis, also on stipes of claviform bodies	Unknown
***Nuraliev et al. 2798*** (Vietnam, Son La) PZ049912	*B. laxiflora* (but most claviform bodies too short)	Unknown	Opposite	Distantly arranged	Spreading	No	Truncate	0.760–0.955	0.280–0.515	Mostly on main axis, also on stipes of claviform bodies	Cream
*Nuraliev, Bragin NUR 4467* (Vietnam, Son La)	*B. laxiflora* (but some claviform bodies too short, and some too wide)	Unknown	Opposite	Distantly arranged	Spreading	No	Truncate	0.767–1.100	0.340–0.824	Mostly on main axis, also on stipes of claviform bodies	Yellow
*Nuraliev et al. 2852* (Vietnam, Son La)	Intermediate between *B. laxiflora* and *B. yakushimensis*	Dioecious	Opposite	Subrosulate	Spreading & erect	No	Convex & truncate	0.650–0.867	0.238–0.432	On main axis and on stipes of claviform bodies	Unknown
*Nuraliev, Kroupsky NUR 4530* (Vietnam, Son La)	Intermediate between *B. laxiflora* and *B. yakushimensis*	Dioecious	Opposite	Distantly arranged	Spreading & erect	No	Truncate	0.564–0.880	0.339–0.562	On main axis and on stipes of claviform bodies	Red
***Nuraliev NUR 4376*** (Vietnam, Thanh Hoa) PZ049916	Intermediate between *B. parajaponica* and *B. yakushimensis*	Unknown	Opposite	Subrosulate	Erect	No	Convex & truncate	0.644–0.845	0.346–0.420	On main axis and on stipes of claviform bodies	Cream
*Nuraliev, Lyskov NUR 3871* (Vietnam, Yen Bai)	Intermediate between *B. laxiflora* and *B. yakushimensis*	Unknown	Likely alternate	Subrosulate	Spreading	No	Convex	1.100–1.400	0.548–0.860	Mostly on main axis, also on stipes of claviform bodies	Unknown

**Figure 1:**
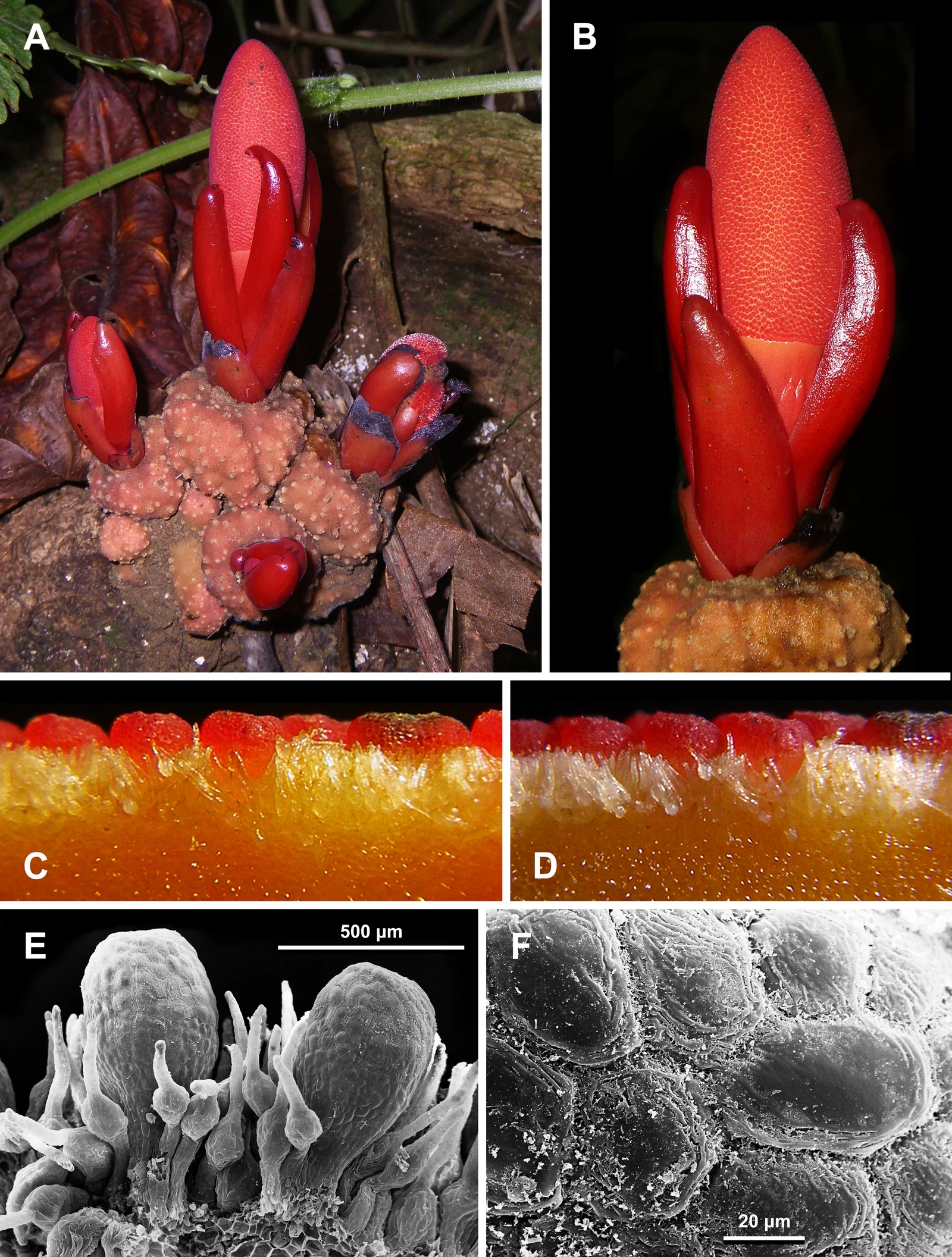
The specimen *Nuraliev NUR 4376*, intermediate between *Balanophora parajaponica* and *B. yakushimensis*. (A) Excavated group of female plants. (B) Female inflorescence. (C, D) Sections of female inflorescence, showing claviform bodies and flowers. (E) Claviform bodies and female flowers, side view (SEM). (F) Epidermal cells at the top of claviform body (SEM). Photos (A–D) Maxim Nuraliev.

**Figure 2:**
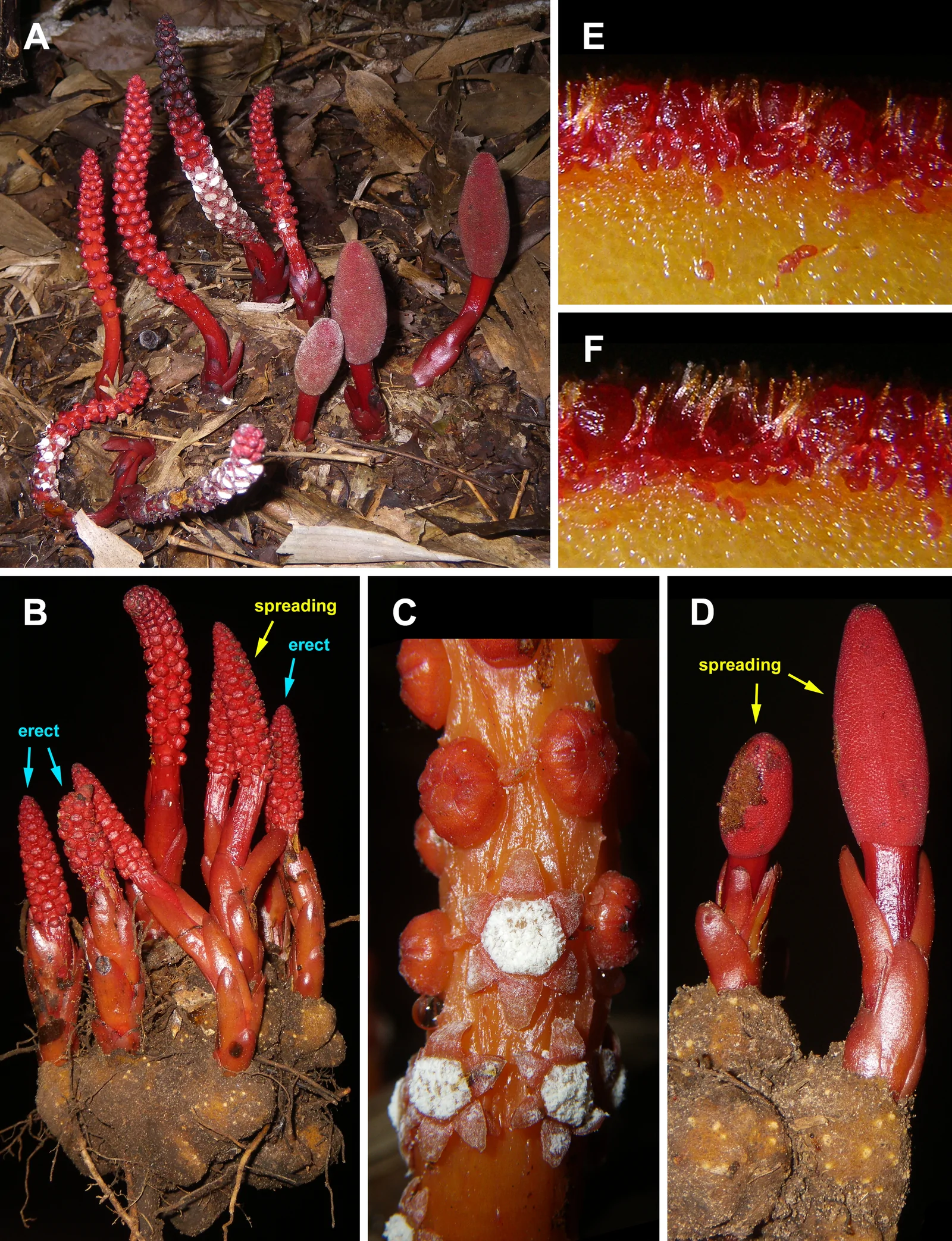
The specimen *Nuraliev, Kroupsky NUR 4530*, intermediate between *Balanophora laxiflora* and *B. yakushimensis*. (A) Group of male and female plants *in situ*. (B) Excavated group of male plants. (C) Portion of male inflorescence. (D) Excavated group of female plants. (E, F) Sections of female inflorescence, showing claviform bodies and flowers. In (B & D) shoots are marked in accordance with presence of spreading or erect leaves on them. Photos: Maxim Nuraliev.

**Table 3:** Cuticle morphology of the claviform bodies in the studied specimens.

Specimen	Shape of cuticle
*Nuraliev et al. G20*	Smooth
*Nuraliev et al. 2282*	With labyrinth-like ridges
*Nuraliev, Lyskov NUR 3771*	With short and labyrinth-like ridges
*Nuraliev et al. 2798*	With labyrinth-like ridges
*Nuraliev NUR 4376*	With short ridges

**Figure 3:**
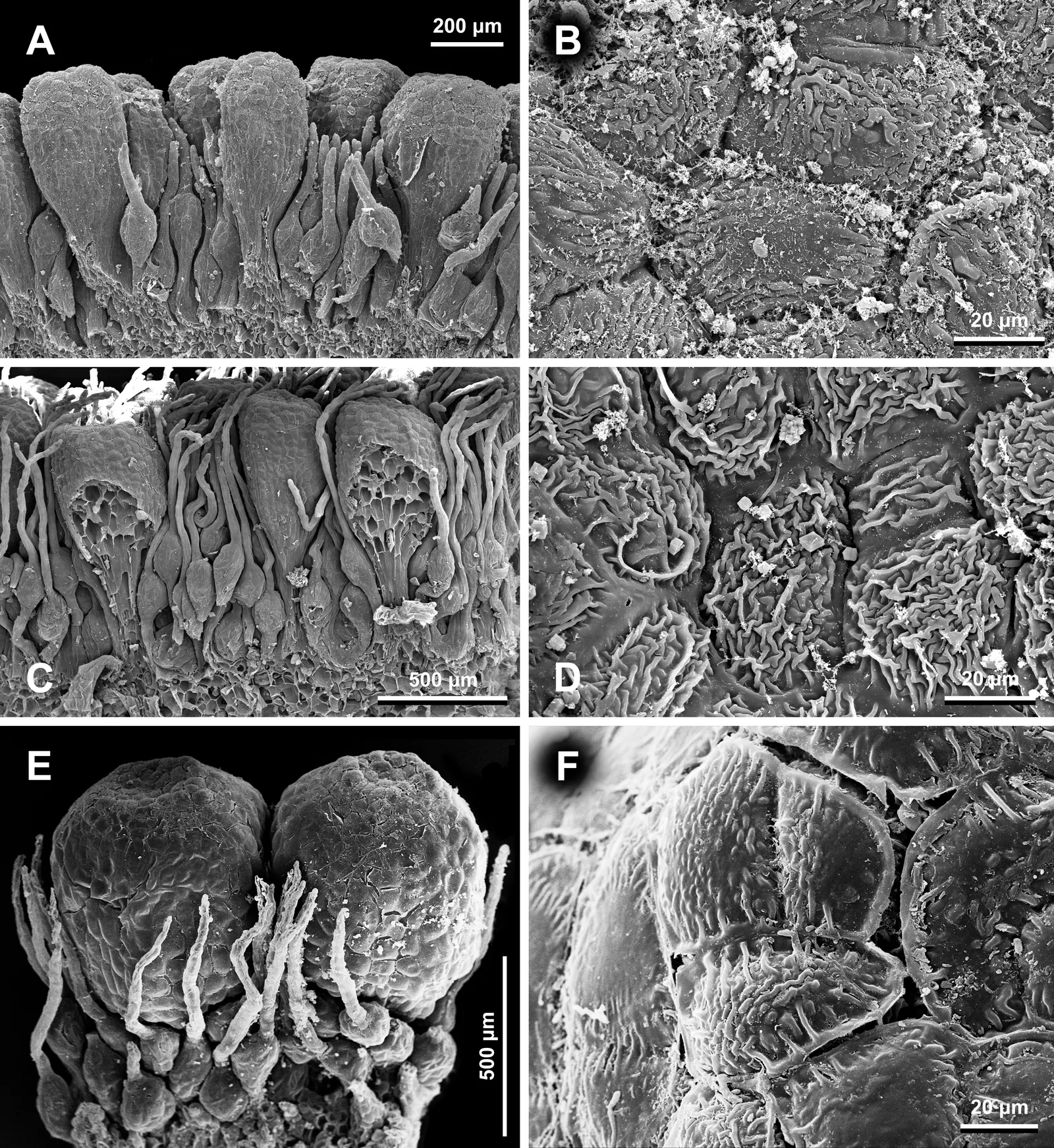
Details of female inflorescences of the representatives of the *Balanophora laxiflora* species complex (SEM): claviform bodies and female flowers, side views (A, C, E) and epidermal cells at the top of claviform body (B, D, F). (A, B) *Nuraliev et al. 2282*. (C, D) *Nuraliev et al. 2798*. (E, F) *Nuraliev, Lyskov NUR 3771*.

**Figure 4:**
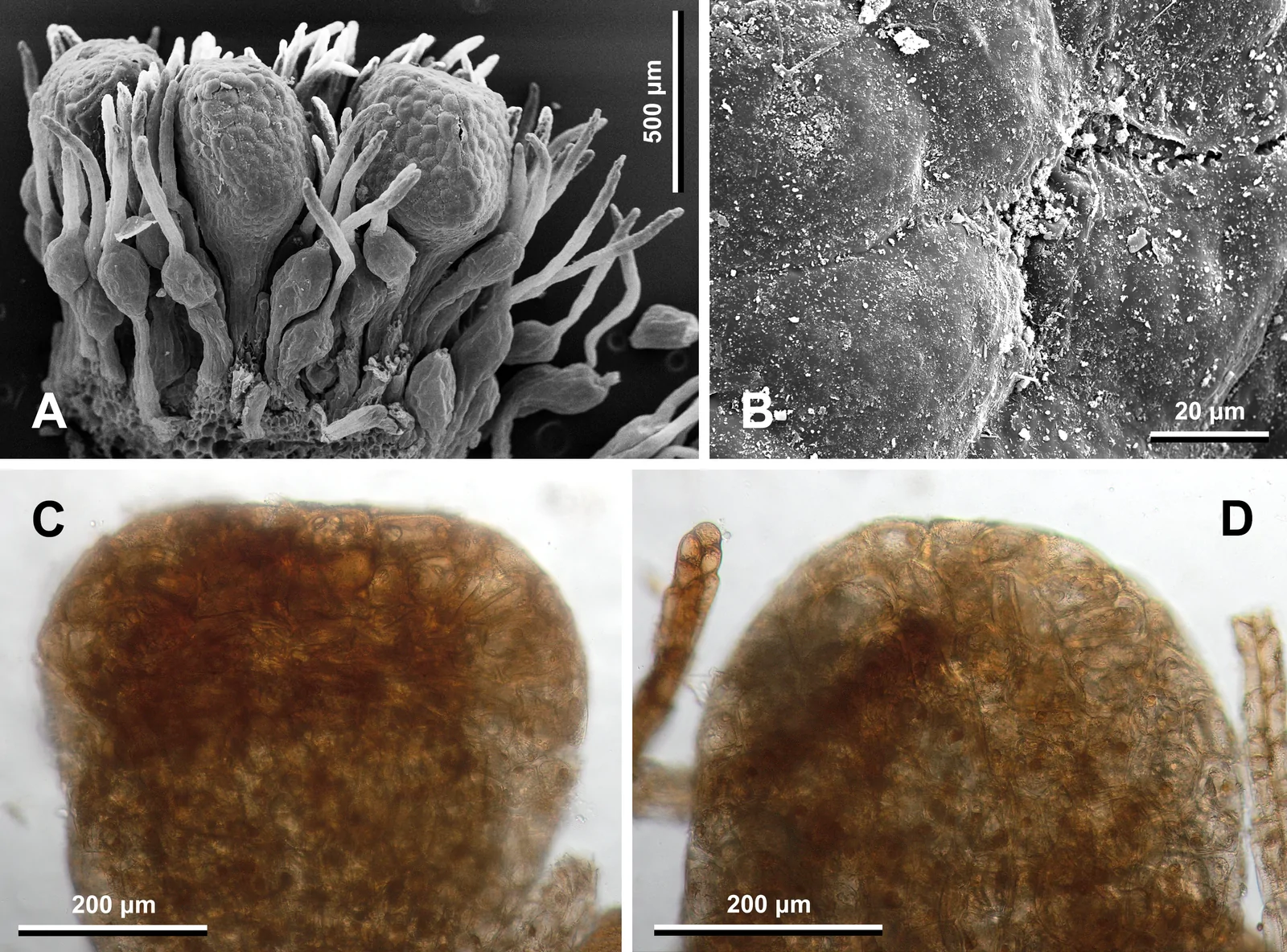
Details of female inflorescences of the representatives of the *Balanophora laxiflora* species complex. (A) *Nuraliev et al. G20*, claviform bodies and female flowers, side view (SEM). (B) *Nuraliev et al. G20*, epidermal cells at the top of claviform body (SEM). (C, D) *Nuraliev et al. 2852*, longitudinal sections of claviform bodies, showing variation in shape (LM).

Among the studied traits, three characters of leaves are invariable at the specimen level: leaf phyllotaxis, leaf arrangement along the stem, and the presence of a thickening at the leaf base. This was also true for the ovary color. In addition, the reproductive system appears to be invariable due to the nature of this trait, as it characterizes the entire population rather than an individual. Leaf posture demonstrated frequent polymorphism (with both spreading and erect conditions found in five of the 16 specimens), and the shape of the apices of the claviform bodies was occasionally polymorphic (with both convex and truncate conditions found in three specimens). The length and width of the claviform bodies often varied broadly within a specimen, as did the arrangement of the female flowers.

### Phylogenetic inference

The aligned set of the 18S rRNA sequences had the following characteristics: initial length, 1,752 bp; length used for phylogenetic inference, 1,747 bp; and 161 variable sites (corresponding to 9% of the alignment). The ML and Bayesian analyses resulted in similar tree topologies; therefore, only the ML tree is presented (Fig. 5).

**Figure 5:**
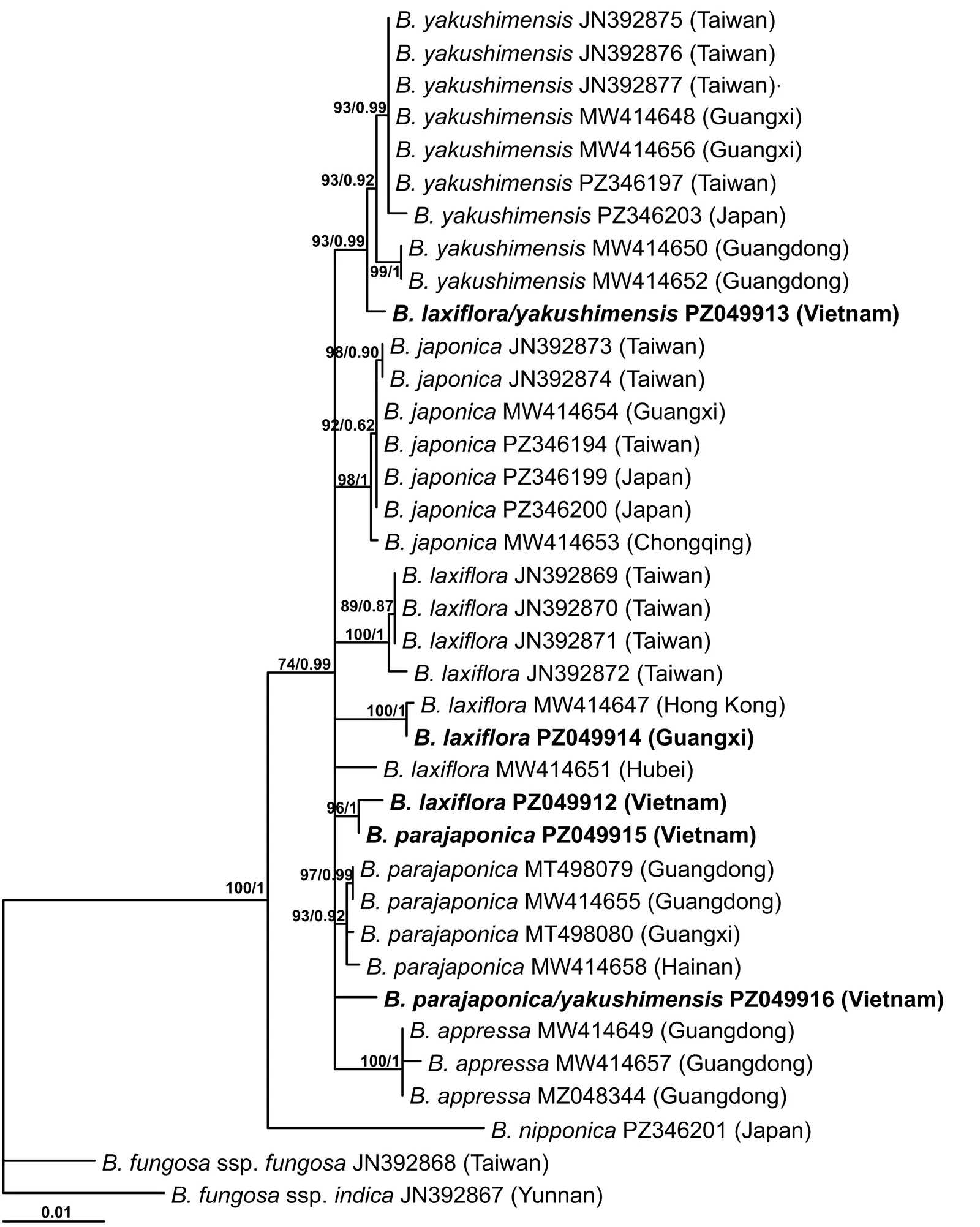
Maximum-likelihood 70% majority-rule consensus tree of the representatives of the *Balanophora laxiflora* species complex inferred from the 18S rRNA gene sequences. The unrooted tree was inferred using IQ-TREE 2 (GTR+I model, 10,000 ultrafast bootstrap replicates); posterior probabilities were assessed using MrBayes 3.2.6 (GTR+I model, 10,000,000 generations, four parallel runs with four Markov chains in each); two accessions of *B. fungosa* were used as an outgroup. Numbers near branches indicate ultrafast bootstrap support values in the maximum-likelihood analysis (UFBoot)/posterior probabilities (PP). The sequences obtained for this study are shown in boldface. Scale bar shows number of substitutions per site.

Within the *Balanophora laxiflora* species complex, *B. nipponica* (represented by a single specimen) is the first lineage to diverge from the remaining accessions, which form a clade supported by an ultrafast bootstrap percentage in the maximum-likelihood analysis (UFBoot) of 74 and a posterior probability in the Bayesian analysis (PP) of 0.99. Relationships within this clade are not fully resolved. Within this polytomy, the following groups can be distinguished:

(1) A clade corresponding to *B. appressa* (UFBoot 100, PP 1.00). It comprises three accessions, namely the type (represented by male and female accession) and a paratype of this species, originating from two different counties of Guangdong, China. These are the only specimens of *B. appressa* included in the analyses.

(2) A clade corresponding to *B. japonica* (UFBoot 98, PP 1.00), *i.e.,* comprising all accessions assigned to this species and no others. These accessions originate from Japan, Taiwan, and Chongqing and Guangxi of China.

(3) A clade (UFBoot 93, PP 0.99) comprising a Vietnamese specimen of uncertain identification (morphologically intermediate between *B. laxiflora* and *B. yakushimensis*), which occupies a sister position to a subclade containing all studied specimens assigned to *B. yakushimensis* (UFBoot 93, PP 0.92). These specimens were collected in Japan, Taiwan, and Guangdong and Guangxi of China.

(4) A clade (UFBoot 100, PP 1.00) consisting of four accessions of *B. laxiflora*. All of these accessions are from Taiwan. Several more specimens of *B. laxiflora* occur outside this clade, but they were collected in other localities.

(5) A clade (UFBoot 93, PP 0.92) consisting of four accessions of *B. parajaponica*, namely its type (represented by male and female accession, from Guangdong) and two paratypes (from Guangxi and Hainan). One more specimen of this species occurs outside this clade.

(6) A clade of two specimens of *B. laxiflora* (UFBoot 100, PP 1.00), one from Guangxi and the other from Hong Kong.

(7) A clade of two specimens (UFBoot 96, PP 1.00), one identified as *B. laxiflora* and the other as *B. parajaponica*. These specimens were collected in distantly located provinces of northern Vietnam. Despite the moderate UFBoot support, this grouping was preferred in the tree topology test, whereas the alternative phylogenetic hypothesis in which the branch uniting the samples was collapsed was rejected at *α* = 0.05 (*p* = 0.001).

In addition, the polytomy includes two individual accessions: one from Hubei, China, identified as *B. laxiflora*, and the other being a Vietnamese specimen morphologically intermediate between *B. parajaponica* and *B. yakushimensis*.

## Discussion

### Overview of the obtained morphological and phylogenetic data

Our study revealed extensive polymorphism of the morphological traits previously used for species delimitation within the *Balanophora laxiflora* species complex. The traits vary independently from each other, further blurring the interspecific boundaries and making the assignment of the specimens to particular species imprecise or even impossible. The phylogenetic reconstructions based on the 18S rRNA gene, although unresolved at some of the nodes, contribute to the indistinctness of the subdivision of the group into species. Indeed, even if the morphologically intermediate specimens are not taken into account, the trees suggest polyphyly of *B. laxiflora* and *B. parajaponica*. The variability of individual characters and its implications for specimen identification and the establishment of phylogenetic species boundaries are discussed in detail below.

### Assessment of individual characters, and their variability

#### Reproductive system

We found that the reproductive system of a specimen of *Balanophora* is difficult to assess when only female individuals are recorded. In the dioecious species of the genus, male and female individuals are sometimes found at some distance from each other, and it is nearly impossible to demonstrate that no male individuals occur in the vicinity of a given female plant (that is, that the population is agamospermous rather than dioecious). For this reason, we scored the reproductive system as unknown in such cases.

#### Leaf phyllotaxis

There is uncertainty regarding the leaf phyllotaxis of *Balanophora japonica*. Although Yu et al. (2021) indicated it as opposite, the leaves were described as “sparse and sometimes subopposite” in the protologue of this species (Makino, 1902), followed by Murata (1999), who stated that they are “spirally arranged or subopposite”. At the same time, a phylogenetic reconstruction with broader sampling (Svetlikova et al., 2026) showed that the specimens studied by Yu et al. (2021) cluster with the other accessions of *B. japonica*, which suggests that the material identification by Yu et al. (2021) was correct. Assuming that Yu et al. (2021) indicated the opposite leaf arrangement based on their own observations rather than by mistake, we treat this as an expansion of the known variability of this character in *B. japonica*.

#### Leaf arrangement along the stem

The arrangement of leaves along the stem, including the length of the internodes between the leaves, had little importance in the taxonomy of *Balanophora* until the studies of Yu et al. (2021) and Yu et al. (2022). In particular, Hansen (1972b) considered this character to be of poor significance for distinguishing closely related species; for example, he synonymized *B. kawakamii* Valeton with *B. harlandii* Hook.f., considering that it differs from typical *B. harlandii* only in having leaves occupying at least half of the stem. Assessment of this character in the *B. laxiflora* species complex generally does not cause difficulties, especially when specimens include mature inflorescences. However, determination of leaf arrangement in young inflorescences that have just begun to flower is quite ambiguous, because their internodes may elongate further and the subrosulate (*i.e.,* rosette-like) arrangement may potentially change into a distant one.

#### Leaves spreading, erect or appressed to the stem

Leaf posture had never been used to distinguish species of the *B. laxiflora* complex until the study of Yu et al. (2021). These authors subdivided variation in leaf posture into three states: spreading, erect and appressed to the stem. As evident from the illustrations provided by Yu et al. (2021), only those leaves were treated as appressed that are tightly connivent to the stem by their entire adaxial surface (in a film-like manner). Erect leaves touch the stem by their margins but not by the adaxial surface (because they are prominently cymbiform), whereas all cases in which the leaves deviate from the stem were assigned to the spreading type.

In our material, leaf posture appeared to vary frequently within a given inflorescence as well as among inflorescences of the same specimen (*e.g.*, Fig. 2). In the former case, we assumed that some leaves have possibly not yet fully bent away from the stem because of immaturity, and therefore scored the leaf posture of the inflorescence as spreading even if only some of the leaves were spreading. In the latter case, we considered the specimen to show polymorphism with respect to leaf posture.

Of the 16 specimens studied here, none have appressed leaves. Eleven specimens possess either spreading or erect leaves, whereas the remaining five specimens are polymorphic, showing spreading leaves in some inflorescences and erect leaves in others.

#### Shape of the apices of the claviform bodies

According to Su, Murata & Hu (2012) and Yu et al. (2021), *Balanophora yakushimensis* is distinct in having the claviform bodies of the female inflorescences with “sunken” apices. This epithet, however, likely resulted from a misinterpretation of the species protologue (Hatusima, 1971), in which the apices of the claviform bodies are described as “obtusis vix depressis” in the Latin diagnosis and as “obtuse scarcely hollowed” in the English note. Thus, the apices of *B. yakushimensis* are to be correctly described as obtuse (*i.e.,* convex) and only slightly depressed, being opposed to the truncate (and sometimes also somewhat concave) apices in the remaining species of the group (Makino, 1902; Makino, 1909; Su, Murata & Hu, 2012; Yu et al., 2021).

In our material, the specimens *Nuraliev et al. 2852* (Figs. 4C, 4D), *Nuraliev, Lyskov NUR 3771* (Fig. 3E) and *Nuraliev NUR 4376* (Figs. 1C–1E) are remarkable in having both truncate and convex claviform bodies within the same inflorescences. They represent the first documented cases of intraspecific variation of this character within the *B. laxiflora* species complex.

#### Arrangement of female flowers

In all the specimens studied here, the female flowers occur both on the main axis of the inflorescence and on the stipes of the claviform bodies. The specimens differ only in the amount of flowers borne on the claviform bodies: the flowers are rather numerous on the claviform bodies in some plants but few there (and therefore mostly arranged on the main axis) in others. The transition between these two variants is remarkably gradual, and in many cases it was difficult to assign an inflorescence to a particular variant. Among the species of the *B. laxiflora* complex, a significantly different morphology is seen in the published illustrations of *B. nipponica* (Makino, 1909: Pl. 3(5); Murata, 1992: Fig. 1) and *B. parajaponica* (Yu et al., 2021: Fig. 4D), in which no flowers are shown on the claviform bodies. However, the text descriptions of both species (Makino, 1909; Yu et al., 2021) state that some flowers do occur on the basal parts of the claviform bodies. Moreover, flowers restricted to the inflorescence axis were depicted in the protologue of *B. hongkongensis* K.M.Lau, N.H.Li & S.Y.Hu (Lau, Li & Hu, 2003: Fig. 1F), which is currently treated as a synonym of *B. laxiflora*. Thus, earlier observations (Makino, 1902; Makino, 1909; Murata, 1990; Murata, 1992; Lau, Li & Hu, 2003; Su, Murata & Hu, 2012; Yu et al., 2021), together with our new data, make it evident that there are no clear differences in the arrangement of the female flowers between the species of the *B. laxiflora* complex, because the species complex demonstrates broad intraspecific variation in this character.

#### Shape of cuticle of the claviform bodies

Hansen (1972b: p. 28) attached great taxonomic significance to the cuticle morphology of the claviform bodies assessed using SEM (see also Murata, 1990). He even developed an identification key to all species of *Balanophora* accepted in his monograph based on the arrangement of the female flowers and the shape of the cuticle. However, the cuticle morphology was rarely analyzed in subsequent taxonomic studies (but see, *e.g.*, Murata, 1990; Murata, 1992; Su, Murata & Hu, 2012).

Within the *B. laxiflora* species complex, cuticle morphology was claimed by Su, Murata & Hu (2012) to be important for distinguishing *B. nipponica* from *B. japonica*, *B. laxiflora* and *B. yakushimensis*. Based on the findings of Murata (1990), Murata (1992) and Murata (1999), Su, Murata & Hu (2012) considered *B. nipponica* to have labyrinth-like cuticular ridges, in contrast to the short ridges present in the latter three species. For *B. appressa* and *B. parajaponica*, this character remains unknown.

Remarkably, Hansen (1972b) reported a smooth cuticle in *B. japonica* (which he circumscribed to include *B. nipponica*), based on the specimen *Murata, Togashi & Kanai 87*, which likely corresponds to *Murata, Togashi & Kanai s.n.* from Miyazaki Prefecture (Kyushu Island, Japan) cited by him among the studied specimens of this species. By contrast, Murata (1990) found distinct short ridges in his specimens from Yakushima Island (Kagoshima Prefecture) and Miyazaki Prefecture attributed by him to *B. japonica s.str.*, as well as conspicuous labyrinth-like ridges in a specimen from Miyagi Prefecture (Honshu Island) assigned to *B. nipponica*. Murata (1990) supposed that the apparently smooth surface observed by Hansen (1972b) resulted from the presence of mucilage covering the claviform bodies, whereas Murata treated the material with bleach to remove the mucilage.

The five specimens studied here demonstrate the entire range of diversity of the cuticle morphology recognized by Hansen (1972b) within the genus *Balanophora*. Notably, the two specimens with exclusively labyrinth-like ridges (*Nuraliev et al. 2282*: Fig. 3B and *2798*: Fig. 3D) occupy sister positions in our phylogenetic reconstructions. The specimen *Nuraliev, Lyskov NUR 3771*, which is sister to the subclade corresponding to *B. yakushimensis*, has some cells with short ridges and others with labyrinth-like ridges (Fig. 3F). Although the non-Japanese species of the *B. laxiflora* complex were believed to uniformly have short ridges, this condition is found in only a single specimen in our study (Fig. 1F). Finally, the remaining specimen shows a smooth cuticle (without any traces of mucilage; Fig. 4B), a condition previously thought to occur only outside the species complex.

As follows from our results, cuticle morphology, similarly to some other characters discussed above, shows not only intraspecific variability, but also variability within a specimen, and even among neighboring cells. Apparently, there is no such strict correspondence between species identity and cuticle shape as previous studies suggested, at least within the *B. laxiflora* species complex.

### Species boundaries in light of the new data

#### Morphological distinction between the species

Species identification of the 16 specimens studied here turned out to be ambiguous for virtually all of them (see Table 2). Information on cuticle morphology of the claviform bodies (obtained for five of the specimens studied here) was not taken into account for specimen identification due to the extensive contradictions outlined above. A significant portion of the uncertainty resulted from the dimensions of the claviform bodies: even when a specimen fits a particular species quite well based on the remaining characters, the length and/or width of the claviform bodies partly or entirely disagrees with the identification. If the dimensions of the claviform bodies are excluded from consideration, only six specimens can be provided with a species name, whereas the remaining ten specimens demonstrate combinations of features that have never been reported for any species of the *Balanophora laxiflora* complex, and therefore they cannot be assigned to any particular species.

Several of the studied specimens were particularly instructive. For example, convex apices of the claviform bodies and red ovaries were considered to be exclusively characteristic of *B. yakushimensis* (Murata, 1999; Yu et al., 2021). However, the specimen *Nuraliev NUR 4376* has convex and truncate apices of the claviform bodies and cream ovaries (see Fig. 1), whereas another specimen, *Nuraliev, Kroupsky NUR 4530*, has truncate apices of the claviform bodies and red ovaries (see Fig. 2). Notably, for some specimens, the reproductive system was scored as unknown (due to the unrecorded male individuals; see above) and/or ovary color was not documented. We believe that, if these data were available, the degree of ambiguity would be even higher, as more specimens would potentially show the combinations of features not known in any of the species.

In addition, considerable differences in the characters assumed to be important for species delimitation were sometimes found among specimens from nearly the same localities (potentially belonging to the same population). For example, the specimens *Nuraliev, Lyskov NUR 3733* and *NUR 3771*, collected at a distance of 400 m from each other, differ in leaf phyllotaxis, the presence of a thickening at the leaf base, and the width of the claviform bodies. Similarly, the specimens *Nuraliev NUR 4239* and *NUR 4244*, with less than 100 m between their localities, differ in the length of the claviform bodies and the arrangement of the female flowers.

In total, four specimens were identified as *B. laxiflora*, one specimen as *B. parajaponica* and one as *B. yakushimensis*, whereas eight specimens show intermediate morphology between *B. laxiflora* and *B. yakushimensis*, one specimen is intermediate between *B. parajaponica* and *B. yakushimensis*, and one is intermediate between *B. japonica* and *B. laxiflora*.

#### Phylogenetic relationships within the species complex

Our phylogenetic reconstructions of the *Balanophora laxiflora* complex do not contradict the idea of distinctness of three of its species, namely *B. appressa*, *B. japonica* and *B. nipponica*. For the remaining three species, there are the following obstacles to consider them monophyletic.

Two specimens included in the phylogenetic study (with GenBank numbers PZ049913 and PZ049916) have uncertain morphology-based identification. Only if one of them is conventionally assigned to *B. yakushimensis* (rather than *B. laxiflora*) and the second to *B. parajaponica* (rather than *B. yakushimensis*), can the monophyly of *B. yakushimensis* be viewed as probable. However, there are no additional grounds for making such decisions.

Then, the close affinity of two accessions from Vietnam, *B. laxiflora* (PZ049912) and *B. parajaponica* (PZ049915), makes both these species non-monophyletic. Species monophyly can be achieved only if the accessions of *B. laxiflora* and *B. parajaponica* are treated as belonging to a single species, and if at the same time the corresponding conventional decisions are made for the two intermediate specimens discussed above.

On the other hand, the issues posed by both the intermediate specimens and the non-monophyletic species can be resolved by merging *B. laxiflora*, *B. parajaponica* and *B. yakushimensis* into a single species. At least, the monophyly of such a consolidated taxon is to be considered possible within the polytomy present in our phylogenetic reconstruction, along with that of Svetlikova et al., 2026: Fig. 5A).

#### Morphological and phylogenetic inconsistencies in light of possible hybridization

Our study revealed high variability in the morphological traits within the *Balanophora laxiflora* species complex that were considered to have diagnostic value at the species level (Yu et al., 2021). Taken together with the intraspecific variation of the 18S rRNA gene found by Svetlikova et al. (2026) in *B. yakushimensis*, this could be viewed as evidence of hybridization. It is particularly interesting that, prior to the inclusion of the Vietnamese samples in the analyses, the morphological and phylogenetic boundaries of the species within the complex were more obvious. It may therefore be supposed that the tropical part of the range of the complex represents a hybrid zone (Abbott, 2017). Three types of hybrid zone have traditionally been recognized: tension, bounded hybrid superiority and mosaic hybrid zones (Abbott, 2017). This classification is based on difference in the fitness of parents and hybrids in different biotopes and niches. Unfortunately, little is known about the ecological characteristics of the lineages within the *B. laxiflora* complex; in particular, no differences in biotope preferences were recorded among the species currently recognized within the complex (Yu et al., 2021).

Given the holoparasitic lifestyle of *Balanophora*, it can be assumed that ecological differences are primarily related to preferences in host plants. Although the existing data indicate a wide host range for individual species of the genus (Hansen, 1972b), Su, Murata & Hu (2012) showed that *B. japonica* and *B. yakushimensis* have distinct host preferences: the former species parasitizes *Symplocos* sp. (Symplocaceae), whereas the latter parasitizes *Distylium racemosum* Siebold & Zucc. (Hamamelidaceae) and *Schima superba* Gardner & Champ. (Theaceae). While these data are encouraging, they cover only two species of the complex and only populations in Japan and Taiwan. The example of *B. yakushimensis* is especially instructive, because its Japanese and Taiwanese populations differ in their hosts despite being confined to a particular host within each of the regions.

The intermediate morphology of some specimens is a weak argument in favor of hybridization, as hybrids do not always exhibit intermediate morphology (Soltis & Soltis, 2009). At the same time, the genus *Balanophora* is generally known for its extraordinarily wide intraspecific variability in almost all morphological traits (Hansen, 1972b). Thus, there is currently no significant evidence for hybridization within the *B. laxiflora* complex. Further detailed studies are needed, with greater emphasis on the ecology and genetic polymorphism of the populations.

#### Possible taxonomic solutions

For determining the number of species within the *Balanophora laxiflora* complex and their boundaries, we follow the concept implying that a species is a phylogenetic lineage (*i.e.,* a monophyletic group of populations); within a group of closely related species, the species usually, but not necessarily, differ morphologically (*e.g.*, De Queiroz, 2007). To this end, we utilize the advantages of an integrative taxonomy approach, analyzing morphological variation within the framework of phylogenetic relationships inferred from DNA data (*e.g.*, Padial & De la Riva, 2010).

As follows from our phylogenetic and morphological findings, one of the possible solutions is the synonymization of *B. parajaponica* and *B. yakushimensis* under *B. laxiflora*. As demonstrated above, only by taking this measure can the non-monophyly of the species within the *B. laxiflora* complex be resolved. No less importantly, this synonymization would remove extensive inconsistencies between the taxonomic outcomes of individual characters and facilitate mostly unequivocal specimen identification. Indeed, the morphological boundaries between the three species were not fully clear prior to our study, and they became even more obscure after consideration of the Vietnamese material.

It should be noted that the species delimitation proposed above (*i.e.,* with inclusion of *B. parajaponica* and *B. yakushimensis* in *B. laxiflora*) does not correspond to the phylogenetic reconstructions obtained by Svetlikova et al. (2026) with employment of plastid sequences. On the one hand, discrepancies are often found when comparing plastome and nuclear sequence phylogenies, and this may be interpreted as a further piece of evidence for hybridization during the evolution of the lineage under study (see above). On the other hand, however, only one plastome of *B. laxiflora* is available for analysis, and data on the plastomes of *B. appressa* and *B. parajaponica* are currently completely lacking. Moreover, the studied conspecific plastomes of *B. japonica* and *B. yakushimensis* apparently do not represent all the lineages identified under these species in the phylogenetic analyses of the 18S rRNA gene sequences. What is particularly important is that not a single Vietnamese specimen is included in any plastome study. Another source of phylogenetic discrepancy, incomplete lineage sorting, which is difficult to distinguish from hybridization, can be invoked to explain the close affinity of the two Vietnamese specimens identified as *B. laxiflora* and *B. parajaponica* inferred in our reconstructions. However, the plastome phylogeny of *Balanophora* similarly cannot be *a priori* taken as a species tree, because plastid genomes are also a subject to incomplete lineage sorting (Doyle, 2022), which means that this phenomenon in either the nuclear or the plastid genome cannot be completely ruled out at the current state of knowledge. For these reasons, we prefer to consider any speculation about the cause of the observed differences (incomplete lineage sorting, hybridization or insufficient sampling) as possible but too premature, at least until a plastome dataset comparable to that of the nuclear 18S rRNA gene is analyzed.

Our clarification of the variability of morphological characters and accurate assessment of previously published data allows discussion of the differences between the species of the *Balanophora laxiflora* complex under various views on the species composition and interspecific boundaries. Even with the boundaries of *B. laxiflora* expanded to include *B. parajaponica* and *B. yakushimensis*, the delimitation of the species complex would remain imperfect from a morphological perspective (Table 4). Indeed, *B. appressa* would appear to be distinct from *B. laxiflora s.l*. solely by its leaves being tightly appressed to the stem, a unique feature of the former species. The only significant distinction previously considered for *B. nipponica*, *i.e.,* the labyrinth-like ridges of the claviform bodies, is no longer valid after our finding of a broad variation in this character irrespectively of species identity (including variation within a given inflorescence). Moreover, *B. japonica* would not show even a single structural difference from *B. laxiflora s.l*. In the key compiled by Yu et al. (2021), *B. japonica* is differentiated from *B. laxiflora* by leaves without a basal thickening, and from *B. parajaponica* by agamospermic reproductive system. However, even in Table 1 of Yu et al. (2021), *B. laxiflora* is described as variable with respect to the presence of a basal thickening of the leaf, which contradicts their key. The reproductive system (*i.e.,* dioecy *vs.* agamospermy), in turn, can hardly be used for species discrimination, because facultative agamospermy is actually widespread in *Balanophora* (Svetlikova et al., 2026), which means that this character is prone to intraspecific variation. The absence of any clear morphological differences between the four species that would remain within the *B. laxiflora* complex after the expansion of *B. laxiflora* discussed here (except for the appressed leaves characteristic of *B. appressa*), combined with their broadly overlapping geographical distribution, would make it necessary to treat them as cryptic species.

**Table 4:** Morphological characteristics of the species of the *Balanophora laxiflora* complex as if *B. parajaponica* and *B. yakushimensis* were merged into *B. laxiflora*.

	*B. appressa*	*B. japonica*	*B. laxiflora* + *B. parajaponica* + *B. yakushimensis*	*B. nipponica*
Source of data	Yu et al. (2021)	Makino (1902), Murata (1999), Yu et al. (2021), Svetlikova et al. (2026)	Hatusima (1971), Yu et al. (2021), Svetlikova et al. (2026), this study	Makino (1909), Murata (1999), Svetlikova et al. (2026)
Reproductive system	Dioecy	Agamospermy	Dioecy with facultative agamospermy	Agamospermy
Leaf phyllotaxis	Alternate	Alternate or opposite	Alternate or opposite	Alternate (to subopposite)
Leaves distantly arranged or subrosulate	Distantly arranged	Subrosulate	Distantly arranged or subrosulate	Distantly arranged
Leaves tightly appressed to stem or not	Yes	No	No	No
Presence of a thickening at leaf base	Yes	No	Yes or no	No
Shape of apices of claviform bodies	Truncate	Truncate	Truncate or convex	Truncate
Shape of cuticle of claviform bodies	Unknown	With short ridges	Smooth, with short ridges or with labyrinth-like ridges	With labyrinth-like ridges
Color of ovary	Cream	Yellow	Yellow to red	Yellow

Our results are remarkably congruent with the ideas of Murata (1990). Although this author followed a narrow species concept due to the interspecific differences he observed in cuticle morphology of the claviform bodies and the arrangement of female flowers, he emphasized the need for thorough chemical and genetic examination to clarify the relationships of *B. japonica*, which he considered morphologically intermediate between *B. nipponica* and *B. yakushimensis*. Similarly, Murata (1990) pointed out the similarity between the female inflorescences of *B. laxiflora* and *B. yakushimensis* and highlighted the necessity of searching for male plants and assessment of their characters in taxonomic studies of this group of species.

Given the poor morphological differentiation of its lineages, it may appear most appropriate to treat the entire *Balanophora laxiflora* clade as a single species. This decision would be fully corroborated by the geographical evidence. Indeed, even the distribution range of *B. laxiflora sensu strictissimo* (*i.e.,* within the limits accepted by Yu et al., 2021 and in the checklist below) nearly coincides with the range of the entire species complex (with the exception of Japan and Hainan). In other words, occurrences of the segregate species are often located nearby populations of *B. laxiflora.* This idea remains speculative until verified by more robust phylogenetic reconstructions with denser sampling.

Thus, our study highlights the need to revise the interspecific boundaries within the *B. laxiflora* complex, most likely by merging at least some of its six currently recognized species. To develop a phylogenetically sound taxonomic concept, further elucidation of the relationships at the base of the *B. laxiflora* clade is required, *e.g.*, through phylogenomic data from an expanded and geographically complete specimen set. In particular, a comparative analysis of phylogenetic reconstructions based on multi-marker nuclear and plastid datasets would help to clarify the issues of incomplete lineage sorting and hybridization. Finally, testing the possibility of crossing between the segregate species can yield valuable results.

## Taxonomic Treatment

### Checklist of the *Balanophora laxiflora* species complex

1. ***Balanophora appressa*** R.X.Yu & S.Y.Zhou, Syst. Bot. 46(4): 1063 (2021)

Distribution: China (Guangdong: type).

2. ***Balanophora japonica*** Makino, Bot. Mag. (Tokyo) 16: 212 (1902)

Distribution: China (Chongqing, Guangxi), Japan: type, Taiwan.

3. ***Balanophora laxiflora*** Hemsl., J. Linn. Soc., Bot. 26: 410 (1894)

= *Polyplethia hexamera* Tiegh., Ann. Sci. Nat., Bot., sér. 9, 6: 195 (1907). ≡ *Balanophora hexamera* (Tiegh.) Lecomte, Bull. Soc. Bot. France 62: 216 (1915). ≡ *Acroblastum hexamerum* (Tiegh.) Setch., Univ. Calif. Publ. Bot. 19: 146 (1935).

= *Balanophora parvior* Hayata, J. Coll. Sci. Imp. Univ. Tokyo 25(19): 192 (1908). ≡ *Balania parvior* (Hayata) Masam., Trans. Nat. Hist. Soc. Formosa 23: 205 (1933). ≡ *Polyplethia parvior* (Hayata) Yamam., J. Soc. Trop. Agric. 5: 406 (1933). ≡ *Acroblastum parvius* (Hayata) Setch. & Yamam., Univ. Calif. Publ. Bot. 19: 146 (1935). ≡ *Polyplethia minor* Nakai, J. Jap. Bot. 15: 748 (1939), *nom. illeg. superfl.*

= *Balanophora spicata* Hayata, J. Coll. Sci. Imp. Univ. Tokyo 25(19): 192 (1908). ≡ *Balania spicata* (Hayata) Masam., Trans. Nat. Hist. Soc. Formosa 23: 205 (1933). ≡ *Acroblastum spicatum* (Hayata) Setch., Univ. Calif. Publ. Bot. 19: 147 (1935). ≡ *Polyplethia spicata* (Hayata) Nakai, J. Jap. Bot. 15: 748 (1939).

= *Balanophora formosana* Hayata, Icon. Pl. Formosan. 3: 168 (1913). ≡ *Balania formosana* (Hayata) Masam., Trans. Nat. Hist. Soc. Formosa 23: 204 (1933). ≡ *Polyplethia formosana* (Hayata) Nakai, J. Jap. Bot. 15: 748 (1939).

= *Balanophora morrisonicola* Hayata, Icon. Pl. Formosan. 5: 198 (1915). ≡ *Polyplethia morrisonicola* (Hayata) Nakai, J. Jap. Bot. 15: 748 (1939).

= *Balanophora oshimae* Yamam., Rep. (Annual) Taihoku Bot. Gard. 1: 96 (1931). ≡ *Polyplethia oshimae* (Yamam.) Yamam., J. Soc. Trop. Agric. 5: 354 (1933).

= *Balanophora rugosa* P.C.Tam, Bull. Bot. Res., Harbin 4(2): 114 (1984).

= *Balanophora taiwaniana* S.S.Ying, Coloured Ill. Fl. Taiwan 5: 514 (1995).

= *Balanophora hongkongensis* K.M.Lau, N.H.Li & S.Y.Hu, Harvard Pap. Bot. 7: 439 (2003).

Distribution: China (possibly Chongqing (type, see Yu et al., 2021), Fujian, Guangdong, Guangxi, Guizhou, Hong Kong, Hubei, Hunan, Jiangxi, Sichuan, Xizang, Yunnan, Zhejiang), Laos, Myanmar, Taiwan, Thailand, Vietnam.

4. ***Balanophora nipponica*** Makino, Bot. Mag. (Tokyo) 23: 59 (1909)

≡ *Balania nipponica* (Makino) Masam., Trans. Nat. Hist. Soc. Formosa 23: 205 (1933). ≡ *Balanophora japonica* var. *nipponica* (Makino) Ohwi, Fl. Jap.: 402 (1965).

= *Balanophora kiusiana* Ohwi, Fl. Jap., rev. ed.: 1437 (1965).

Distribution: Japan: type.

5. ***Balanophora parajaponica*** R.X.Yu, S.Y.Zhou & Y.Q.Li, Syst. Bot. 46(4): 1063 (2021)

Distribution: China (Guangdong: type, Guangxi, Hainan), Vietnam (but see notes in Table 2).

Taxonomic note: there is evidence for synonymization of this species with *B. laxiflora*.

6. ***Balanophora yakushimensis*** Hatus. & Masam., J. Geobot. 19(3): 61 (1971)

Distribution: China (Guangdong, Guangxi), Japan: type, Taiwan, Vietnam (but see notes in Table 2).

Taxonomic note: there is evidence for synonymization of this species with *B. laxiflora*.

### Examined specimens

(see Table 2 for detailed comments on identification)

**CHINA.**

Guangdong Province, Shenzhen city, Dapeng Peninsula Geopark, western slope of Qiniang Mountain, forest, 22°31′32.9″N 114°32′17.3″E, elev. 547 m, 30 November 2023, *Yu.O. Kopylov-Guskov, E.E. Severova CIF177* (MW, stored in ethanol);

Guangxi Zhuang Autonomous Region: Liuzhou City, Luzhai County, Xiangqiao Karst National Geological Park, limestone slope, 24°44′57″N 109°39′40″E, elev. 180 m, 20 November 2016, *M.S. Nuraliev, N.A. Vislobokov, F. Wen, Z.B. Xin G20* (MW, stored in ethanol).

**VIETNAM.**

Cao Bang Province: Nguyen Binh District, Phia Oac-Phia Den National Park, 10 km SW of Nguyen Binh town, bamboo forest, 22°35′39″N 105°52′51″E, elev. 1,200 m, 7 October 2018, *M.S. Nuraliev, A.N. Kuznetsov, S.P. Kuznetsova 2282* (MW, stored in ethanol);

Ha Giang Province: Vi Xuyen District, Cao Bo municipality, Tay Con Linh Nature Reserve, disturbed forest with tea plantations on slope, 22°45′21″N 104°51′02″E, elev. 780 m, 16 October 2019, *M.S. Nuraliev, Yu.O. Kopylov-Guskov, D.F. Lyskov 2925* (MW, stored in ethanol);

Lang Son Province: Cao Loc District, Mau Son municipality, Mau Son special-use forest, 21 km E of Lang Son city, trail to Nui Phia Me summit, forest with bamboo on slope, 21°50′55″N 106°58′47″E, elev. 1,140 m, 28 September 2024, *M.S. Nuraliev NUR 4239* (MW, stored in ethanol);

Lang Son Province: Cao Loc District, Mau Son municipality, Mau Son special-use forest, 21 km E of Lang Son city, trail to Nui Phia Me summit, forest with bamboo on slope, 21°50′55″N 106°58′47″E, elev. 1,140 m, 28 September 2024, *M.S. Nuraliev NUR 4244* (MW, stored in ethanol);

Lao Cai Province: Bat Xat District, Bat Xat Nature Reserve, 4 km SSE of Y Ty village, local ridge, forest with bamboo, 22°37′13″N 103°37′01″E, elev. 2,010 m, 17 October 2018, *M.S. Nuraliev 2334* (MW, stored in ethanol);

Lao Cai Province: Bat Xat District, Trung Leng Ho municipality, Bat Xat Nature Reserve, trail to Ky Quan San Mountain, forest on slope, 22°30′17″N 103°36′46″E, elev. 2,230 m, 5 November 2022, *M.S. Nuraliev, D.F. Lyskov NUR 3733* (MW, stored in ethanol);

Lao Cai Province: Bat Xat District, Trung Leng Ho municipality, Bat Xat Nature Reserve, trail to Ky Quan San Mountain, near tourist camp, forest, 22°30′26″N 103°36′57″E, elev. 2,110 m, 8 November 2022, *M.S. Nuraliev, D.F. Lyskov NUR 3771* (MW, stored in ethanol);

Lao Cai Province: Sa Pa District, Hoang Lien National Park, Cat Cat village area, foot of hill, on slope, not far from river, 22°19′25″N 103°49′50″E, elev. 1,250 m, 26 November 2009, *M.S. Nuraliev NUR 179a* (MW, stored in ethanol);

Son La Province: Bac Yen District, Ta Xua Nature Reserve, disturbed forest with bamboo, near stream, 21°18′43″N 104°31′37″E, elev. 1,380 m, 5 October 2019, *M.S. Nuraliev, Yu.O. Kopylov-Guskov, D.F. Lyskov 2798* (MW, stored in ethanol);

Son La Province: Bac Yen District, Hang Dong commune, Ta Xua Nature Reserve, 21.325179°N 104.524229°E, 12 October 2025, *M.S. Nuraliev, A.M. Bragin NUR 4467* (MW, stored in ethanol);

Son La Province: Phu Yen District, Ta Xua Nature Reserve, disturbed forest with bamboo, 21°21′01″N 104°40′39″E, elev. 510 m, 8 October 2019, *M.S. Nuraliev, Yu.O. Kopylov-Guskov, D.F. Lyskov 2852* (MW, stored in ethanol);

Son La Province: Van Ho District, Chieng Xuan commune, Xuan Nha Nature Reserve, forest with bamboo on slope, 20.694173°N 104.710293°E, elev. 1,070 m, 23 October 2025, *M.S. Nuraliev, I.A. Kroupsky NUR 4530* (MW, dried specimen and also stored in ethanol);

Thanh Hoa Province: Ba Thuoc District, Thanh Son municipality, near Pu Luong Nature Reserve, 18 km NW of Canh Nang town, vicinities of Pu Luong Mountain, disturbed forest with bamboo, 20°28′16″N 105°06′35″E, elev. 830 m, 18 October 2024, *M.S. Nuraliev NUR 4376* (MW, stored in ethanol);

Yen Bai Province: Mu Cang Chai District, Cao Pha commune, area of Huy Thanh (Khau Pha) fish farm, disturbed forest, steep slope, near stream, 21°44′17″N 104°16′38″E, elev. 1,120 m, 19 November 2022, *M.S. Nuraliev, D.F. Lyskov NUR 3871* (MW, stored in ethanol).

## Conclusions

Our study shed new light on the morphological diversity and phylogenetic structure of the *Balanophora laxiflora* species complex, one of the most taxonomically controversial lineages of *Balanophora*, which has been accepted during the last several years as consisting of six species. Expansion of the sampling through the inclusion of populations from the Indochinese Peninsula resulted in documentation of previously unknown combinations of morphological traits, which made it impossible to assign such specimens to any species within the current taxonomic system of the complex. In addition, the specimens studied here often demonstrated broad ranges of variation of one or another character, crossing the assumed interspecific boundaries. In line with the morphological findings, the phylogenetic placement of some Vietnamese specimens based on the nuclear 18S rRNA gene indicated that the subdivision of the complex into six species partly contradicts the idea of species monophyly.

These contradictions can be removed only through the application of a broader species concept to the *B. laxiflora* complex. The strongest contradictions concerned a group of three species, *B. laxiflora*, *B. parajaponica* and *B. yakushimensis*, which are differentiated neither morphologically nor phylogenetically. Consequently, one of the possible solutions is to treat them as a single species, *i.e., B. laxiflora s.l*. This decision would also be supported by geographical evidence, as *B. parajaponica* and *B. yakushimensis* have been reported largely within the distribution range of *B. laxiflora s.str*. After this rearrangement, the *B. laxiflora* complex would be subdivided into four species, each potentially corresponding to a phylogenetic lineage. At the same time, our data demonstrate that such four species would still be virtually indistinguishable from each other by morphological traits, corresponding to the concept of cryptic species. Thus, further research may show that the most appropriate taxonomic approach is to treat the entire species complex as a single species, *B. laxiflora sensu latissimo*.

Our results illustrate that the full complexity of a broadly distributed and highly variable lineage can be uncovered only through large-scale analysis with stepwise focusing on different geographical areas and morphological traits. In particular, they underscore the importance of thorough evaluation of morphological features at each level, with assessment of a sampling of individuals per population, several inflorescences per individual, and a selection of flowers and other parts from different portions of an inflorescence. Similarly, elucidation of phylogenetic structure within a lineage requires employment of a geographically representative set of accessions.

## Supplemental Information

10.7717/peerj.21913/supp-1 Supplemental Information 1 Raw data: Alignment of the 18S rRNA sequences used in this study

10.7717/peerj.21913/supp-2 Supplemental Information 2 Raw data: Primers for amplification of the 18S rRNA gene used in this study

## Additional Information and Declarations

### Field Study Permissions

The following information was supplied relating to field study approvals (*i.e.*, approving body and any reference numbers): The Joint Vietnam-Russia Tropical Science and Technology Research Center approved the study (1037, 2225, 2750, 3302, 3535, 5110, 5464, 5861).

### Data Availability

The following information was supplied regarding data availability: The data is available in the Supplemental Files. The data is available at Zenodo: Zavialov, A., Samigullin, T., Lyskov, D., Fomichev, C., Logacheva, M., & Nuraliev, M. (2026). Species composition in the Balanophora laxiflora species complex (Balanophoraceae): molecular and morphological evidence [Dataset]. Zenodo. https://doi.org/10.5281/zenodo.20746763
The sequence data are available at NCBI: PZ049912–PZ049916.
