# Supplementary material for: Species composition in the Balanophora laxiflora species complex (Balanophoraceae): molecular and morphological evidence
Source: PeerJ. 2026 Nov 2;14:e21913. doi: 10.7717/peerj.21913 (PMC13738192; doi:10.7717/peerj.21913)
Supplement: Supplementary file 1 [file peerj-14-21913-s002.pdf]

Primers for amplification of the 18S rRNA gene used in this study (designed by *Su, Murata & Hu, 2012*).

18S12f TCCTGCCAGTASTCATATGC
18S540f TGGTGCCAGCAGCCGC
18S1050f GGTCGCAAGGCTGAAACT
18S555r GCGGCTGCTGGCACCA
18S1065r AGTTTCAGCCTTGCGACC
18S1769r CACCTACGGAAACCTTGTT
